# Association Between Gestational Diabetes Mellitus and Maternal Depression: A Narrative Review

**DOI:** 10.7759/cureus.86886

**Published:** 2025-06-27

**Authors:** Kalyani Rath, Smitha MV

**Affiliations:** 1 Obstetrics and Gynaecology, Kalinga Institute of Nursing Sciences, Bhubaneswar, IND; 2 College of Nursing, All India Institute of Medical Sciences, Bhubaneswar, Bhubaneswar, IND

**Keywords:** gestational diabetes mellitus, glycemic control, maternal depression, perinatal health, psychological support

## Abstract

Gestational diabetes mellitus (GDM) and maternal depression are increasingly recognized as interrelated conditions that significantly impact maternal and neonatal outcomes. This narrative review synthesizes current evidence on the bidirectional relationship between GDM and maternal depression, emphasizing their combined burden on perinatal health. GDM, characterized by glucose intolerance identified during pregnancy, is experiencing a rising global prevalence and is linked to an increased risk of depressive symptoms both during pregnancy and postpartum. Women diagnosed with GDM show a markedly higher incidence of depression compared to their non-GDM counterparts, influenced by factors such as metabolic dysregulation, inflammatory pathways, and the psychosocial stressors associated with managing a high-risk pregnancy. Moreover, maternal depression in the context of GDM is associated with suboptimal glycemic control, a higher incidence of obstetric complications, and diminished maternal quality of life. Fetal risks, including preterm birth, abnormal birth weight, and adverse neurodevelopmental outcomes, are heightened when maternal depression coexists with GDM. Biological mechanisms, such as dysregulation of the hypothalamic-pituitary-adrenal axis, insulin resistance, and chronic inflammation, provide plausible connections between these conditions. The evidence highlights the necessity for integrated, multidisciplinary care models that incorporate routine mental health screenings within GDM management protocols. Early identification and intervention for depressive symptoms, along with patient education and psychosocial support, are critical to improving clinical outcomes. This review advocates for holistic, culturally sensitive approaches and calls for further longitudinal research to elucidate the underlying mechanisms and enhance perinatal care strategies that address both the metabolic and psychological aspects of maternal health.

## Introduction and background

Diabetes is primarily a lifestyle condition that has increased alarmingly across all age groups in India, and the prevalence among the younger population has increased above 10%. India is now referred to as the “Diabetes Capital of the World” [1], as it accounts for 17% of the total number of diabetes patients in the world. Diabetes is a significant global health issue where currently close to 80 million people with diabetes are in India, and this number is expected to increase to 135 million by 2045 [2].

Gestational diabetes mellitus (GDM) is defined as a form of hyperglycemia or glucose intolerance that is first recognized or diagnosed during pregnancy. It typically manifests as elevated blood glucose levels that do not meet the criteria for overt diabetes but are above normal pregnancy glucose thresholds [3].

The prevalence of GDM has been rising steadily due to various factors, including increasing rates of obesity, sedentary lifestyles, and advanced maternal age. According to the International Diabetes Federation (IDF), in 2019, approximately 23 million women aged 20-79 were living with diabetes, a category that encompasses GDM. This number is projected to rise to 343 million by 2045. The IDF Diabetes Atlas estimates that in 2021, 21.1 million live births were affected by hyperglycemia in pregnancy, with 80.3% of these cases attributed to GDM [4]. In particular, hyperglycemia in pregnancy affects 20 million women annually, which accounts for 16% of all live births, with 84% of these cases attributed to gestational diabetes. Globally, one in six births is affected by gestational diabetes, highlighting the scale of the issue [5].

Depressive disorder, commonly referred to as depression, is a prevalent mental health condition. It is characterized by a persistent low mood or a diminished interest or pleasure in activities for extended periods. Depression is distinct from normal mood fluctuations and everyday emotional responses. It can influence all facets of life, impacting relationships with family, friends, and the wider community [6].

An estimated 3.8% of the population struggles with depression, which includes 5% of adults (4% among men and 6% among women) and 5.7% of adults over the age of 60. Approximately 280 million people worldwide are affected by this condition [7]. Depression is about 50% more prevalent in women than in men. Research indicates that more than 10% of pregnant women and those who have recently given birth may experience symptoms of depression on a global scale [8].

Maternal depression refers to a range of depressive conditions that can affect mothers during pregnancy and up to 12 months postpartum. This spectrum includes prenatal depression, postpartum depression (PPD), and postpartum psychosis. Recognized as a significant public health concern globally, maternal depression can have profound effects on various aspects of a woman's life, including her work, family dynamics, and the health and development of her child [9].

Maternal depression is a critical public health concern that carries profound implications for both maternal and infant health. It has been associated with an increased risk of obstetric complications, as well as long-term developmental and psychological challenges for the child. If left unaddressed, maternal depression can lead to lasting impairments in cognitive, emotional, and behavioral outcomes in offspring, underscoring the need for early detection and intervention [10].

GDM has been increasingly recognized as a contributing factor to maternal depression, with the physical, emotional, and hormonal challenges of GDM potentially heightening a woman's vulnerability to psychological distress during and after pregnancy.

The global prevalence of GDM in pregnant women is estimated to be approximately 14%. Research indicates that the incidence of depression among pregnant women diagnosed with GDM varies between 25.9% and 56.7%. Additionally, some studies have found that as many as 32% of women with GDM experience depression during their pregnancy [11].

Recent studies have revealed a notable link between GDM and maternal depression, especially PPD. A systematic review and meta-analysis involving 18 studies with over 2.3 million participants found that GDM considerably raised the risk of PPD, with a pooled relative risk of 1.59 (95% CI: 1.22-2.07) [12,13].

Emerging evidence suggests that the connection between GDM and depression is bidirectional. A prospective cohort study discovered that women with GDM had a 4.62 times greater risk of developing postpartum depressive symptoms. In contrast, early pregnancy depression was linked to a heightened risk of developing GDM, indicating that depressive symptoms might play a role in triggering GDM [14,15].

The relationship between GDM and depression varies across different populations. A meta-analysis focused on low- and middle-income countries indicated that women with GDM had almost double the odds (OR: 1.92) of experiencing perinatal depression (PND) compared to those without GDM. This emphasizes the need for context-specific interventions and screenings in various settings [16].

The relationship between GDM and maternal depression is intricate and multifaceted. The study aims to emphasize the relationship between GDM and maternal depression, which highlights the need for integrated healthcare approaches that consider both metabolic and mental health dimensions during pregnancy and after childbirth.

## Review

Search methodology

The search methodology for this narrative review followed a comprehensive and structured approach to identifying relevant literature. The review utilized two commonly used electronic databases, PubMed and Web of Science. Search terms included “gestational diabetes,” “maternal depression,” and related keywords, combined using Boolean operators to refine and optimize search results.

**Figure 1:**
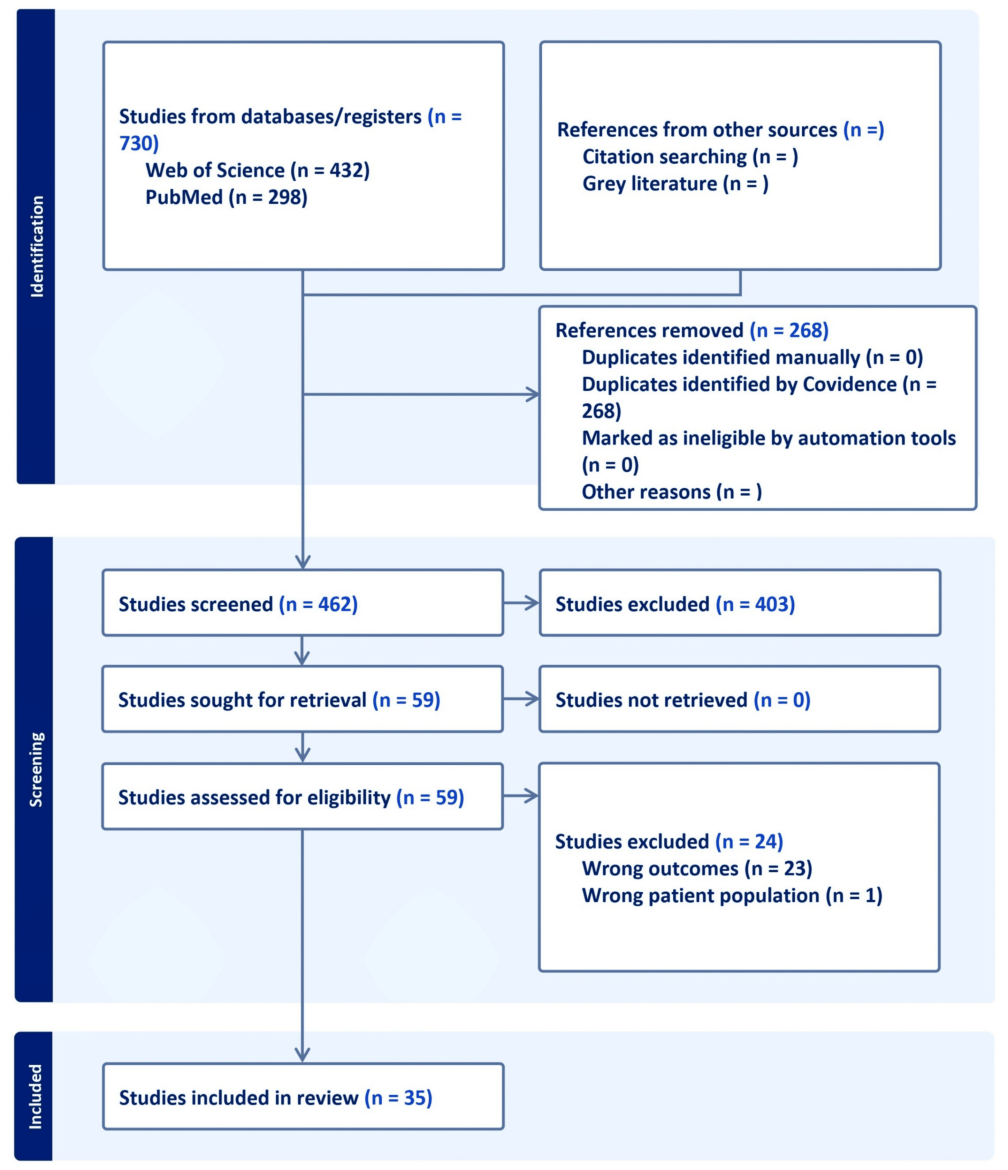
Search strategy

The inclusion criteria focused on studies examining both the direct and indirect associations between GDM and maternal depression. This review includes studies involving pregnant women diagnosed with GDM, with an assessment or diagnosis of maternal depression either during pregnancy (antenatal) or postpartum. Eligible studies include observational designs (cohort, case-control, and cross-sectional), clinical studies, review articles, and qualitative studies exploring maternal experiences. Only studies reporting on depression symptoms, clinical diagnosis, or mental health outcomes in the context of GDM were considered. Articles had to be published in English, fall within the 2015-2025 publication window, and be available in full text. A total of 730 studies were identified using the specified search terms across the databases. All articles were screened for relevance, quality, and methodological rigor. Of these, 35 studies specifically examined the association between GDM and maternal depression and were deemed relevant for inclusion in this narrative review. These studies were thoroughly reviewed to assess the strength of the association, explore epidemiological evidence, and evaluate the impact on both the mother and fetus (Figure 1).

Epidemiological evidence linking GDM and maternal depression

The overall prevalence of antenatal depression was found to be 18.32%, reflecting a substantial mental health burden among pregnant women. Stratification by GDM status revealed a marked difference: depression was significantly more prevalent among women with GDM (25.92%) compared to those without GDM (10.38%). This finding underscores the growing body of evidence suggesting a strong association between GDM and maternal depression. A higher burden of depression has been reported among women with GDM, particularly among primiparous women, who may experience greater psychological stress due to the combined impact of a first pregnancy and a high-risk diagnosis. The added psychological vulnerability in first-time mothers may stem from a lack of prior pregnancy experience and increased anxiety related to fetal health, especially when compounded by a diagnosis of GDM [17].

Depression symptoms were more pronounced during the second trimester, affecting 23.4% of women with GDM compared to a significantly lower prevalence among non-GDM subjects. This suggests that the second trimester may be a critical window for the onset or exacerbation of depressive symptoms, coinciding with intensified metabolic changes and clinical management of GDM [18].

The timing of GDM diagnosis introduces additional complexity. Early-onset GDM was identified in 20.4% of women, while late-onset GDM occurred in 19.3%. Notably, women with early-onset GDM had a higher prevalence of depression (20.9%) compared to those with late-onset GDM (15.6%, p = 0.06). Although this difference narrowly missed being statistically significant, the trend indicates that earlier exposure to the physical and emotional challenges of GDM may raise the risk of depression [19]. Additionally, early pregnancy screening showed that 14.9% of women exhibited depressive symptoms and 17.5% reported anxiety, underscoring the importance of early psychosocial evaluations during antenatal care. These overlapping mental health concerns emphasize the interconnected nature of depression, anxiety, and metabolic disorders in pregnancy.

GDM had a 54% higher risk of developing PPD compared to non-GDM counterparts (adjusted hazard ratio [aHR] 1.54; 95% CI: 1.42-1.67) [20]. Women with a history of depression were more likely to develop GDM, indicating a bidirectional relationship between the two conditions.

The pathophysiological mechanisms linking GDM and depression are still being elucidated. However, shared pathways such as chronic low-grade inflammation, dysregulation of the hypothalamic-pituitary-adrenal (HPA) axis, insulin resistance, and altered neurotransmitter function have been proposed. Additionally, the psychosocial impact of managing a high-risk pregnancy, including dietary restrictions, medication adherence, and concern over fetal complications, may further heighten emotional distress.

Association between GDM and maternal depression

The interplay between GDM and maternal depression is increasingly recognized as a significant concern in perinatal health. Both conditions are known to independently contribute to adverse maternal and neonatal outcomes. Understanding their co-occurrence is essential for developing targeted interventions and ensuring comprehensive care.

GDM increases the risk of peripartum depression, and that depressive symptoms during pregnancy may also contribute to poor metabolic control. A bidirectional relationship exists where metabolic and psychological processes may reinforce each other [21]. Factors such as glycemic control, social support, and lifestyle behaviors play a significant role in influencing PPD risk among women with GDM, which underscores the importance of early psychological assessment and support [22].

Diabetes mellitus in pregnancy (DMP), including GDM, is associated with a higher risk of PPD. Women diagnosed with DMP had more than twice the odds of developing PPD compared to those without, even after adjusting for confounding factors such as maternal age, body mass index, parity, and a history of depression [23]. Furthermore, the combination of DMP and antepartum depression was associated with an exceptionally high risk of PPD, suggesting that metabolic and psychological stressors may act synergistically.

Educational age, glycosylated hemoglobin, parity, and GDM were associated with depression, with educational age and parity as protective factors, and glycosylated hemoglobin and GDM as risk factors [22].

Women who develop diabetes during pregnancy are at increased risk of experiencing PPD, suggesting that metabolic disturbances may contribute to adverse mental health outcomes. Conversely, poor mental health in early pregnancy has been linked to a higher likelihood of developing GDM, particularly among women from low socioeconomic backgrounds [23,24]. Despite a high prevalence of poor mental health indicators in this group, no statistically significant difference in GDM incidence was observed between women with and without these symptoms. It is suggested that high baseline levels of psychosocial stress in low socioeconomic settings may obscure more subtle associations that could be more apparent in diverse or general populations.

The biological mechanisms potentially linking GDM and maternal depression include dysregulation of the HPA axis, chronic systemic inflammation, and insulin resistance [25]. Elevated levels of cortisol and inflammatory biomarkers such as C-reactive protein (CRP) and interleukin-6 (IL-6), common to both conditions, may contribute to both hyperglycemia and depressive symptoms. Furthermore, the emotional and logistical burden of managing a pregnancy complicated by GDM may itself exacerbate depressive symptoms, reinforcing a potentially cyclical relationship.

The link between GDM and maternal depression is complex, shaped by factors such as the timing of diagnosis, evaluation criteria, socioeconomic status, and coexisting health conditions. Integrated screening and treatment strategies address both mental health and metabolic health issues simultaneously during the antenatal period.

Impact of maternal depression among GDM mothers

Maternal Outcomes

Maternal depression concurrent with GDM significantly impacts the physical and psychological well-being of affected women. Evidence indicates that depression during pregnancy increases the risk of developing GDM, and once diagnosed, women with GDM are more likely to experience worsening depressive symptoms. This combination can lead to poorer glycemic control, complicating diabetes management and increasing pregnancy-related complications [26,27]. Additionally, depression among women with GDM has been linked to reduced breastfeeding self-efficacy during pregnancy and postpartum, potentially undermining maternal confidence and health behaviors [28]. The coexistence of depression and GDM has also been associated with lower postpartum quality of life [27]. Moreover, recent studies from 2018 and 2020 emphasize that maternal depression in GDM mothers is related to increased anxiety, stress, and impaired adherence to prenatal care recommendations, which further deteriorate maternal outcomes [29,30].

Fetal Outcomes

Maternal depression in the context of GDM is associated with several adverse fetal outcomes. Pregnancies complicated by both depression and GDM exhibit increased risks of preterm birth and low birth weight, which are linked to neonatal morbidity [27]. PND combined with GDM also elevates the likelihood of macrosomia and neonatal hypoglycemia due to poor maternal glycemic control and stress-related hormonal changes affecting fetal growth [31]. Newer research from 2017 and 2021 further suggests that elevated maternal cortisol levels caused by depression may negatively impact fetal neurodevelopment and increase risks for metabolic disorders in offspring later in life [31-33]. These findings highlight the critical need for integrated care addressing both mental health and metabolic control to optimize fetal health outcomes.

Public Health Implications

The growing association between DMP, particularly GDM, and PND underscores critical public health considerations. Evidence from recent studies suggests that women with GDM are at a significantly increased risk of developing depressive symptoms during both the antenatal and postpartum periods, indicating a bidirectional relationship between metabolic and mental health during pregnancy [23,34-36].

This relationship has clear implications for public health policy and clinical practice. First, existing screening frameworks for perinatal mental health should be expanded to include GDM as a significant risk factor. Universal depression screening in pregnant individuals diagnosed with GDM would enable earlier identification and intervention, potentially reducing the burden of untreated PND and its associated complications [23,35].

Furthermore, educational interventions for women diagnosed with GDM should go beyond glycemic control to include information on the recognition of depressive symptoms, the impact of stress on glucose regulation, and strategies for emotional resilience. Empowering women with this knowledge may improve not only mental health outcomes but also adherence to diabetes management regimens [36].

Integrated care models are essential. The incorporation of mental health services into routine obstetric and endocrinologic care for women with GDM can enhance care coordination and improve outcomes. Such models should include psychosocial support, cognitive-behavioral strategies, and routine psychological assessments to address the complex needs of this population [36].

Finally, broader public health strategies must include targeted campaigns to raise awareness about the mental health risks associated with GDM, particularly in underserved or high-risk populations. This may include the development of culturally sensitive community health programs and digital health tools to improve access to education and support services.

In summary, the evidence indicates a compelling need to reframe the management of GDM through a multidisciplinary lens that prioritizes both physical and mental health. Addressing PND risk among GDM mothers through early detection, education, and integrated care may lead to improved maternal well-being and healthier developmental outcomes for offspring (Table 1).

**Table 1:** Features of review articles GDM, gestational diabetes mellitus; PPD, postpartum depression; LMICs, low- and middle-income countries.

Authors	Year	Key Findings	Conclusion
Alzarooni KI et al. [12]	2024	Identified predictive factors for perinatal depression in women with GDM	Key psychosocial and medical predictors outlined
Han M et al. [13]	2019	Examined neuromedin U levels in diabetes	No correlation with insulin secretion or GDM
Dias JM et al. [14]	2025	Studied stress and postpartum depression in GDM patients	Stress significantly contributes to postpartum depression in GDM
Jin Y et al. [16]	2024	Meta-analysis on GDM and perinatal depression in LMICs	GDM significantly raises risk of perinatal depression
Natasha K et al. [17]	2015	Hospital-based study in Bangladesh on depression and GDM	Higher depression prevalence found in GDM patients
Munda A et al. [18]	2021	Longitudinal study on depression and anxiety in GDM	GDM linked with increased depressive and anxiety symptoms
Hemavathy S et al. [19]	2025	Study of depression/anxiety in pregnant women with GDM in South Asia	High prevalence confirmed across the region
Kim H et al. [20]	2020	Population-based cohort on GDM and perinatal depression	Confirmed association between GDM and depression
Žutić M et al. [21]	2024	Longitudinal study on GDM and peripartum depression relationship	GDM and depression influence each other
Tan J et al. [22]	2024	Factors influencing postpartum depression in GDM patients	Identified biological and psychosocial risk factors
Ross GP et al. [23]	2016	Systematic review on depression and diabetes in pregnancy	Strong evidence linking diabetes and maternal depression
Liu Y et al. [24]	2017	Meta-analysis on maternal depression and early childhood development	Maternal depressive symptoms negatively impact child development
Duarte-Guterman P et al. [25]	2019	Inflammation's role in postpartum depression	Inflammation may contribute to postpartum depression
Miller NE et al. [26]	2021	Impact of GDM diagnosis on concurrent depression	GDM diagnosis may increase depression risk
Azami M et al. [27]	2019	Systematic review on GDM and postpartum depression	Strong association found between GDM and postpartum depression
Işık G et al. [28]	2022	GDM’s effect on depression and breastfeeding self-efficacy	GDM linked to depression and lower breastfeeding confidence
Clevesy MA et al. [29]	2018	Relationship between GDM and perinatal depression	Potential link found; further studies needed
Atraki S et al. [30]	2020	Anxiety and depressive disorders in diabetic pregnant women	High mental health burden in diabetic pregnancies
Kampmann U et al. [31]	2015	Clinical update on GDM	Reviews diagnosis, management, and risks of GDM
Damm P et al. [32]	2016	Long-term GDM effects on mother and child in Denmark	GDM has lasting health impacts
Nath A et al. [33]	2017	Maternal stress and cortisol linked to infant development in India	Stress adversely affects infant cognitive outcomes
Wilson RE et al. [34]	2020	Review of GDM and perinatal mental health	Increased risk of mental disorders in women with GDM
Jung S et al. [35]	2021	Psychosocial support for GDM women	Such interventions are effective and beneficial
Huang T et al. [36]	2015	Pregnancy hyperglycemia and depression risk	Hyperglycemia increases risk for depression
Hinkle SN et al. [37]	2016	Longitudinal study on depression and GDM	Observed two-way relationship between GDM and depression
Miller ES et al. [38]	2016	Diabetes association with postpartum depression	Diabetes raises the risk of postpartum depression
Damé P et al. [39]	2017	LINDA-Brazil study on GDM and depressive symptoms	High rates of depressive symptoms in women with GDM
Lee KW et al. [40]	2019	Mental health burden in Malaysian women with GDM	High levels of depression, anxiety, and stress reported
Özkahraman-Koç S et al. [41]	2019	GDM's effect on anxiety, depression, and prenatal attachment	GDM negatively impacts maternal emotional health and attachment
Li H et al. [42]	2022	Longitudinal cohort study on GDM and perinatal depression	Strong GDM-depression link across the perinatal period
Tasnim S et al. [43]	2022	Pilot study on antenatal depression in women with GDM	Elevated depression prevalence confirmed
Huang S et al. [44]	2022	Chinese study on depression determinants in GDM	Identified social and health-related predictors
Singh AK et al. [45]	2023	GDM and postpartum depression in Eastern India	Significant association confirmed
Yamada K et al. [46]	2023	Depression and diet-related distress in Japanese GDM patients	Dietary management linked to distress and depression
Björvang RD et al. [47]	2024	Systematic association of diabetes in pregnancy and perinatal depression	Conclusive evidence of increased depression risk in GDM pregnancies

Recommendations for policy and practice

The connection between GDM and maternal depression emphasizes the importance of integrated health policies and clinical practices. It is vital to include regular mental health screenings in prenatal care for women with GDM to ensure early identification and intervention. Tools like the Edinburgh Postnatal Depression Scale (EPDS) and Patient Health Questionnaire (PHQ-9) should be utilized throughout pregnancy and after childbirth, recognizing GDM as a significant psychosocial risk factor. Prioritizing interdisciplinary care that involves obstetric, endocrine, and mental health professionals is essential, along with improved patient education regarding the psychological effects of GDM. Healthcare provider training programs must incorporate mental health management related to GDM, backed by systemic investments in integrated care services. Additionally, using electronic health records can enhance care coordination. National maternal health strategies should integrate mental health into universal coverage frameworks in line with global maternal health objectives. Continued research and focus on health disparities are essential for improving maternal and neonatal outcomes.

## Conclusions

In conclusion, the evidence presented in this narrative review underscores the multifaceted and bidirectional relationship between GDM and maternal depression. Both conditions individually pose significant risks to maternal and fetal health, but their co-occurrence exacerbates adverse outcomes, including poor glycemic control, reduced adherence to prenatal care, compromised maternal quality of life, and unfavorable neonatal outcomes such as preterm birth and impaired neurodevelopment. The psychosocial burden associated with managing GDM, combined with hormonal and inflammatory changes, appears to heighten vulnerability to depressive symptoms during pregnancy and postpartum.

Due to the increasing prevalence of both GDM and maternal depression worldwide, especially in low- and middle-income countries, there is an urgent need for integrated clinical and public health strategies that cater to the physical and psychological needs of pregnant women. Routine mental health screening in GDM care, interdisciplinary collaboration, targeted education, culturally sensitive interventions, and systemic support for healthcare professionals must become standard components of maternal healthcare. Furthermore, the implementation of longitudinal research and policies tailored to sociocultural and economic contexts will be essential to effectively mitigate the dual burden of GDM and maternal depression. A holistic, person-centered approach to perinatal care is crucial for improving maternal mental health and ensuring optimal outcomes for both mothers and their children.
